# Gleanings from Our Exchanges

**Published:** 1873-06-15

**Authors:** 


### Gleanings from Our Exchanges.

Action of Cold Water on the Spleen.—The importance which many authors, and Fleury especially, have ascribed to cold water as a remedial agent in intermittent fever and various other affections of the spleen, has induced Prof. Mosler, of Greifswalde, to test its efficacy, and he has arrived at the following conclusions:

1. The direct contact of cold water with the normal spleen causes it to contract appreciably, and the degree of contraction varies according to the temperature of the water and the length of time for which it is employed.
2. Cold water also acts similarly on the normal spleen, if applied over the abdominal walls, but it is less effectual. In such cases the cold douche is to be preferred to cold compresses or bits of ice, but in all of them it accomplishes less than quinine.
3. In all forms of enlarged spleens, the result of disease, whether acute or chronic, the action of cold water causes contraction of the organ.
4. The paroxysms of intermittent fever can be arrested by Fleury's method of using the cold douche, but it has no advantage over quinine, either in new or old cases.
5. The cold douche does not accomplish a permanent cure of this fever. It does not always prevent recurrence, and leaves the spleen enlarged.
6. The spleen of typhoid fever is diminished in size by the same remedy.
7. In all forms of enlargement of the spleen, whether acute or chronic, the application of cold water to the spleen, either in the form of cold baths, ice-bags, or cold douches, with the internal use of quinine at the same time, is to be preferred to either of these methods alone.
8. As a substitute for quinine in chronic cases, quinoidine is recommended as being very much cheaper.—*Archiv f. path. anat.*, lvii., 1.—*Medical Record.*

Balsam of Copaiba in Small-Pox and Scarlatina (*Medical Times and Gazette*, February 17).—From our knowledge of the effects of the balsam on the skin and mucous membranes, Dr. A. Rowland, Visiting Physician and Surgeon to the Marine and Emigrant Hospital, etc., Quebec, was induced to try it, in four or five drop doses, mixed in two drachms syrup and two drachms mucilage gum of Arabic, three or four times a day, in the confluent small-pox of a person who had never been vaccinated. It caused no nausea, but, on the contrary, created a keen appetite, which continued till recovery. No pitting took place, and no local application was used but glycerine and water. Dr. Rowland tried the same mixture in scarlet fever, with most satisfactory results. Under its use, the tongue and sore throat got rapidly clean and well, followed by a keen appetite, and by none of the usual sequelæ. The secretion of urine was copious, and began to increase in quantity after two or three doses. At first it was the color of ale and a little ropy, but by the third day quite clear and normal. The author's theory of the action of the remedy is, that it alters or destroys the character of the virus, and eliminates it out of the system by the skin and kidneys more particularly; for the recoveries have been unusually rapid. In both cases Dr. Rowland prescribed milk, beef-tea, wine and spirits, according to need.—*Southern Med. Record.*

Is Hard Water Wholesome?—Dr. Letheby, after devoting many years to an investigation into the properties of the water introduced into English cities, and to a study of the sanitary reports on the subject, comes to the conclusion that moderately hard water is safer and more healthful than soft water. Hard water is not only clearer, colder, more free from air, and consequently more agreeable to the eye and to the taste than soft water, but it is less likely to absorb organic substances, to sustain the life of zymodic organisms, or to exert solvent properties upon salts of iron or upon leaden conducting pipes. The lime salts exert a beneficial influence upon the animal economy, and even protect the system from dangerous outward influences. Dr. Wilson, of Edinburgh, has also collected much valuable material on the subject, and comes to the same conclusions as Dr. Letheby. He takes the ground that the human body requires for its nourishment and support a supply of mineral salts, among which carbonate and phosphate of lime play an important part in building up the compactness of the bones, and in other functions.—*Med. and Surg. Rep.*

Sulphate of Iron in Suppuration.—A child burned nearly over the whole body was recently brought to the Child's Hospital at Lausanne. The suppuration was so abundant that the ward in which the patient lay became uninhabitable. The child was then placed in a bath containing two handfuls of sulphate of iron. The cessation of pain was almost immediate. After repeating the bath twice a day, for fifteen or twenty minutes at a time, the suppuration moderated, the fœtid odor disappeared, and the patient rapidly recovered.—*Boston Journal of Chemistry.*

Distribution of Consumption.— The volume of "Vital Statistics of the Census" is provided with colored charts, four of which show the distribution of disease in the United States. The blue chart denotes the ratio of deaths by consumption, the darker tints assuming a wedge shape, roughly defined by lines drawn from Chattanooga, on the one hand through Richmond, and on the other through St. Louis. Most in the shadow of this dreadful disease are the New England coast, all of New Hampshire and Northern Vermont. Malarial diseases (yelow chart) haunt the Atlantic and Gulf coast, from Delaware Bay to Texas, while New England and the larger Middle States, with the two Virginias, are comparatively free from them. California, which, in the central portion, is tolerably consumptive, is universally malarial, but in a light degree.—*Medical and Surgical Reporter.*

Nephrotomy.—W. W. Dawson, M.D., of Cincinnati, Ohio (*N. Y. Med. Jour.*) reports a case of extraction of a calculus from the kidney of a married woman, aged 50 years, who had borne nine children, and had always been a healthy and robust woman until eight years ago, when she had an attack of hæmaturia. The patient died on the fifth day. The calculus was quite light, weighing only 20¾ grains, and measuring seven-eighths of an inch in length by half an inch in breadth at its widest part, being uniform in shape. It was ammoniac magnesian phosphate, and situated on the internal wall at a point corresponding to the hilus of the kidney, and was embedded in partly disorganized fibrin. The kidney-tissue which he incised was about one-half inch in thickness, and bled but little.—*Med. Rec.*

Treatment of Diseases of the Bladder by the Injection of Urine.—Dr. Clemens, of Frankfort, uses healthy urine, containing the normal amount of uric acid, as an injection into the bladder in cases of catarrh. The organ should be first cleaned from any fœtid urine and washed out by the injection of tepid water ; a healthy young subject is then got to pass urine into the syringe, which has been previously warmed, and then it is injected into the diseased bladder. The best results are obtained where the urine of healthy boys and of adults is used on alternate occasions. In one instance a single injection of healthy urine sufficed to allay a spasm, and in others the injection used twice or three times a day, produced a most beneficial effect.—*Med. and Surg. Rep.*

Pill for Gastralgia.—℞. Sub. nitrate of bismuth, ℥ ii.; ext. belladonna, gr. x. Make into forty pills, give one night and morning.

Spina - Bifida.—Dr. Wilson, of Glasgow, relates a case of spina-bifida which he treated thus: The tumor being moistened over with carbolized oil, was opened by a free longitudinal incision, under an antiseptic veil of surgeon's lint soaked in carbolized oil. The lint was removed, and a large piece of carbolized plaster applied, and over this another, kept in position by adhesive plaster, one edge being left comparatively free for escape of fluid. A soft folded handkerchief was laid over all. A good firm pad of skin resulted.

Hydrate of Chloral in Incontinence of Urine.—Dr. Girolamo Leonardi has found chloral a most valuable remedy in nocturnal incontinence of urine. The dose for children is from five to ten grains taken in water before going to bed. For adults the dose is proportionately larger. The treatment has been successful in all of his recorded cases. The remedy must be repeated for several successive nights.—*Lo Sperimentale*, April, 1873.

Large Child.—At a recent meeting of the Obstetrical Society of Boston, Dr. Minot said he had lately attended a lady whose child, when dressed, weighed but a trifle less than fifteen pounds !

A knowledge of Greek is no longer compulsory to matriculants in the University of London. A candidate may pass in any one of the three languages, Greek, French, or German.

Lactic Acid in Dyspepsia.—Dr. C. Handfield Jones advises the use of latic acid in dyspepsia. He has chiefly given it in cases of irritative dyspepsia, where the digestion was painful and imperfect, and had been so for some time ; he does not advise its use at the commencement of the treatment in a severe case, but only after irritation and vascular erethism is somewhat reduced. It should be employed in doses of fifteen or twenty minims in a half ounce of water, and taken *at* meal-times ; he states that it seems to mingle with the food, and to supply one of the constituents of healthy gastric juice, which is probably imperfectly produced. Its use need not be confined to cases of dyspepsia, but may be extended to all cases where it is desirable to improve the tone and power of the stomach. It is pleasant, occupies but little space, and the only objection to its use is its present high price ; but if much employed, it could probably be obtained cheaply.—*Southern Medical Record.*

Vermifuge Syr.—Extract spigelia maryl, fl ℥ viii.; syr. sennæ compos. (Lond. Ph.) ℔. ii. Mix while hot, and evaporate to a proper consistence. Dose, a small teaspoonful for a child one year old.

PHILADELPHIA, June 2, 1873.

*Messrs. Editors:*—The editorials of the Philadelphia *Medical Times*, and the N. Y. *Medical Record*, on the session of the American Medical Association, forcibly reminded one of the editorials which so frequently met our view during the late war, written by men who stayed at home and told others who exposed themselves in the field how to fight.

Yours, A. M. A.

